# Oxygenation laryngoscope vs. nasal standard and nasal high flow oxygenation in a technical simulation of apnoeic oxygenation

**DOI:** 10.1186/s12873-021-00407-5

**Published:** 2021-01-22

**Authors:** H. Herff, W. A. Wetsch, S. Finke, F. Dusse, T. Mitterlechner, P. Paal, V. Wenzel, D. C. Schroeder

**Affiliations:** 1 Department of Anaesthesiology and Intensive Care Medicine, University of Cologne, Faculty of Medicine and University Hospital of Cologne, Kerpener Str. 67, 50937 Cologne, Germany; 2 Department of Anaesthesiology, Privatklinik Hochrum, Sanatorium der Kreuzschwestern, Rum, Austria; 3 Department of Anaesthesiology and Intensive Care Medicine, Hospitallers Brothers Hospital, Paracelsus Medical University Salzburg, Salzburg, Austria; 4 Department of Anaesthesiology, Intensive Care Medicine, Emergency Medicine and Pain Therapy, Klinikum Friedrichshafen, Friedrichshafen, Germany

**Keywords:** Apnoeic oxygenation, Oxygenation laryngoscope, High flow nasal oxygenation, Oxygen desaturation, Technical simulation

## Abstract

**Background:**

Failed airway management is the major contributor for anaesthesia-related morbidity and mortality. *Cannot-intubate-cannot-ventilate* scenarios are the most critical emergency in airway management, and belong to the worst imaginable scenarios in an anaesthetist’s life. In such situations, apnoeic oxygenation might be useful to avoid hypoxaemia. Anaesthesia guidelines recommend careful preoxygenation and application of high flow oxygen in difficult intubation scenarios to prevent episodes of deoxygenation. In this study, we evaluated the decrease in oxygen concentration in a model when using different strategies of oxygenation: using a special oxygenation laryngoscope, nasal oxygen, nasal high flow oxygen, and control.

**Methods:**

In this experimental study we compared no oxygen application as a control, standard pure oxygen application of 10 l·min^− 1^ via nasal cannula, high flow 90% oxygen application at 20 l·min^− 1^ using a special nasal high flow device, and pure oxygen application via our oxygenation laryngoscope at 10 l·min^− 1^. We preoxygenated a simulation lung to 97% oxygen concentration and connected this to the trachea of a manikin model simulating apnoeic oxygenation. Decrease in oxygen concentration in the simulation lung was measured continuously for 20 min.

**Results:**

Oxygen concentration in the simulation lung dropped from 97 ± 1% at baseline to 40 ± 1% in the no oxygen group, to 80 ± 1% in the standard nasal oxygen group, and to 73 ± 2% in the high flow nasal oxygenation group. However, it remained at 96 ± 0% in the oxygenation laryngoscope group (*p* < 0.001 between all groups).

**Conclusions:**

In this technical simulation, oxygenation via oxygenation laryngoscope was more effective than standard oxygen insufflation via nasal cannula, which was more effective than nasal high flow insufflation of 90% oxygen.

## Background

Failed airway management is the major contributor for anaesthesia-related morbidity and mortality [1, 2]. *Cannot-intubate-cannot-ventilate* scenarios are the most critical emergency in airway management, and belong to the worst imaginable scenarios in an anaesthetist’s life. As failure in airway management cannot be excluded in every patient, preventing the patient from desaturation is essential to have enough time for alternative airway management procedures.

Apnoeic oxygenation might be a useful tool to prolong the time before desaturation occurs in difficult ventilation situations [3]. Accordingly, the guidelines for management of difficult airway scenarios suggest careful preoxygenation and the application of a “bulk flow of oxygen” [4]. In the last few years, tools have been developed to maintain high gas flows, delivering an oxygen/air mixture via nasal cannula [5]. We have previously reported a newly designed dual-use laryngoscope [6], which may be an alternative system suitable for gas delivery into the deep laryngeal space in an unexpectedly difficult ventilation scenario in order to maintain apnoeic oxygenation. Based on this and other studies, some authors postulated that the use of oxygenation laryngoscopes for the management of difficult airways might be an alternative to high flow oxygen insufflation devices [7]. However, the oxygenation laryngoscope and high flow oxygen insufflation devices have not previously been compared.

In this study, we evaluated the decrease in oxygen concentration in a model when using different strategies of oxygenation. The formal hypothesis was that there would be no difference in oxygen concentration decrease in the simulation lung between the groups.

## Methods

As this was designed as a technical simulation without any participants, no approval from the Ethics Committee was required.

**Fig. 1:**
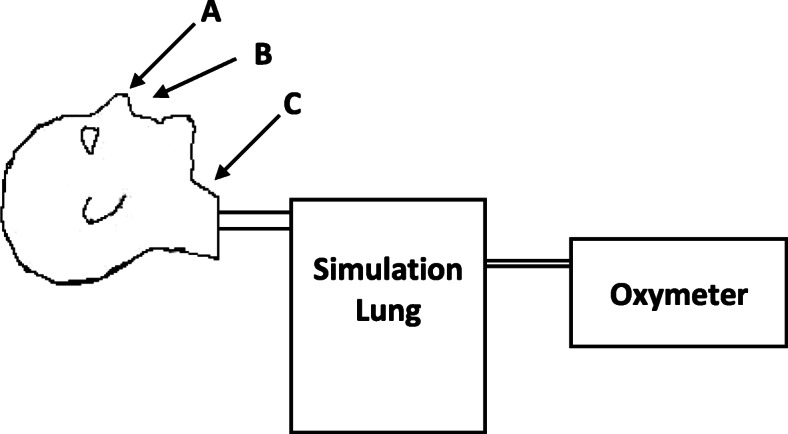
Schematic of experiment with placement of oxygen source: **a** Standard flow nasal oxygen, **b** High flow oxygenation device via nasal cannula, **c** oxygenating laryngoscope

We used a standard airway manikin (Laerdal® Adult Airway Management Trainer, Laerdal Medical, Stavanger, Norway) with an attached standardized simulation lung (capacity 2.5 l), which is comparable to the functional residual capacity of an adult male. At the base of the simulation lung, a paramagnetic oximeter (Primus, Dräger, Lübeck, Germany) with an accuracy of ±(2.5 Vol% + 2.5 rel.) was attached (Fig. 1). Sampling was performed at a suction rate of 200 ml·min^− 1^. This value is comparable to oxygen consumption during apnoea in adults [6].

We compared decreases in oxygen concentration in an experimental setting in a preoxygenated simulation lung attached to a manikin model for four different strategies of oxygen insufflation. The first strategy was no oxygen application to provide a baseline. The second strategy was standard oxygen application via nasal cannula. The third was oxygen application via a special nasal cannula using a high flow oxygen insufflation device, and the fourth was via the oxygenating laryngoscope.

**Fig. 2:**
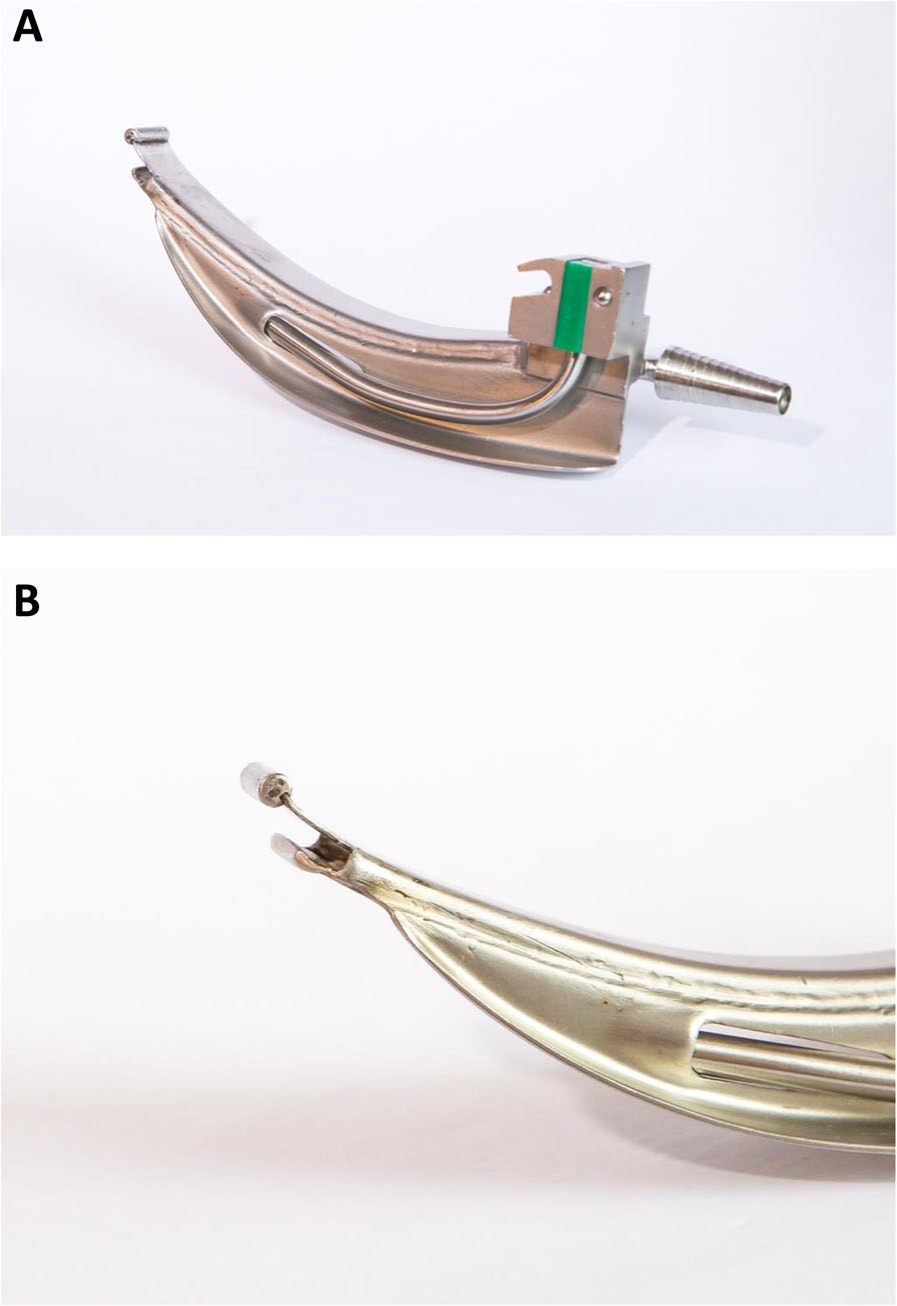
**a + b**. Prototype of our oxygenating laryngoscope, that provides an internal lumen to provide oxygen insufflation into the deep laryngeal space. From dorsal side view (**a**) and close view of the oxygen channel ending at the top of the laryngoscope (**b**)

In the control group, there was no oxygen insufflation. In the standard oxygen via nasal cannula group we applied oxygen at 10 l·min^− 1^ (Flowmeter single, Heyer Aerotech, Nieven, Germany). In the high flow group we applied an air-oxygen mixture (90% oxygen/10% nitrogen) at 20 l·min^− 1^ using an Airvo2 (Fisher&Paykel Healthcare Ltd., Auckland, New Zealand) connected to the flowmeter with maximum flow (approx. 15 l·min^− 1^). In the oxygenation laryngoscope group we applied 10 l·min^− 1^ oxygen via the oxygenating laryngoscope *(*Fig. 2*a+b)*.

After preoxygenating the simulation lung to 97% oxygen concentration, we performed five experiments in random order for each of the four groups, continuously measuring the oxygen content in the simulation lung for 20 min. Data is reported as mean ± standard deviation.

After Kolmogorov-Smirnov analysis, a one-way analysis of variance for repeated measurements was performed to determine the overall statistical significance between the four groups, followed by post hoc Student’s Newmann-Keuls test for pairwise multiple comparisons (Sigmaplot 14; Systat, San Jose, CA). *p* < 0.05 was considered as being significant.

## Results

Oxygen content in the simulation lung dropped from 97 ± 1% at baseline to 40 ± 1% in the no oxygen group, to 80 ± 1% in the nasal insufflation group, to 73 ± 1% in high flow nasal system group, and to 96 ± 0% in the oxygenation laryngoscope group (*p* < 0.009 between all groups).

**Fig. 3:**
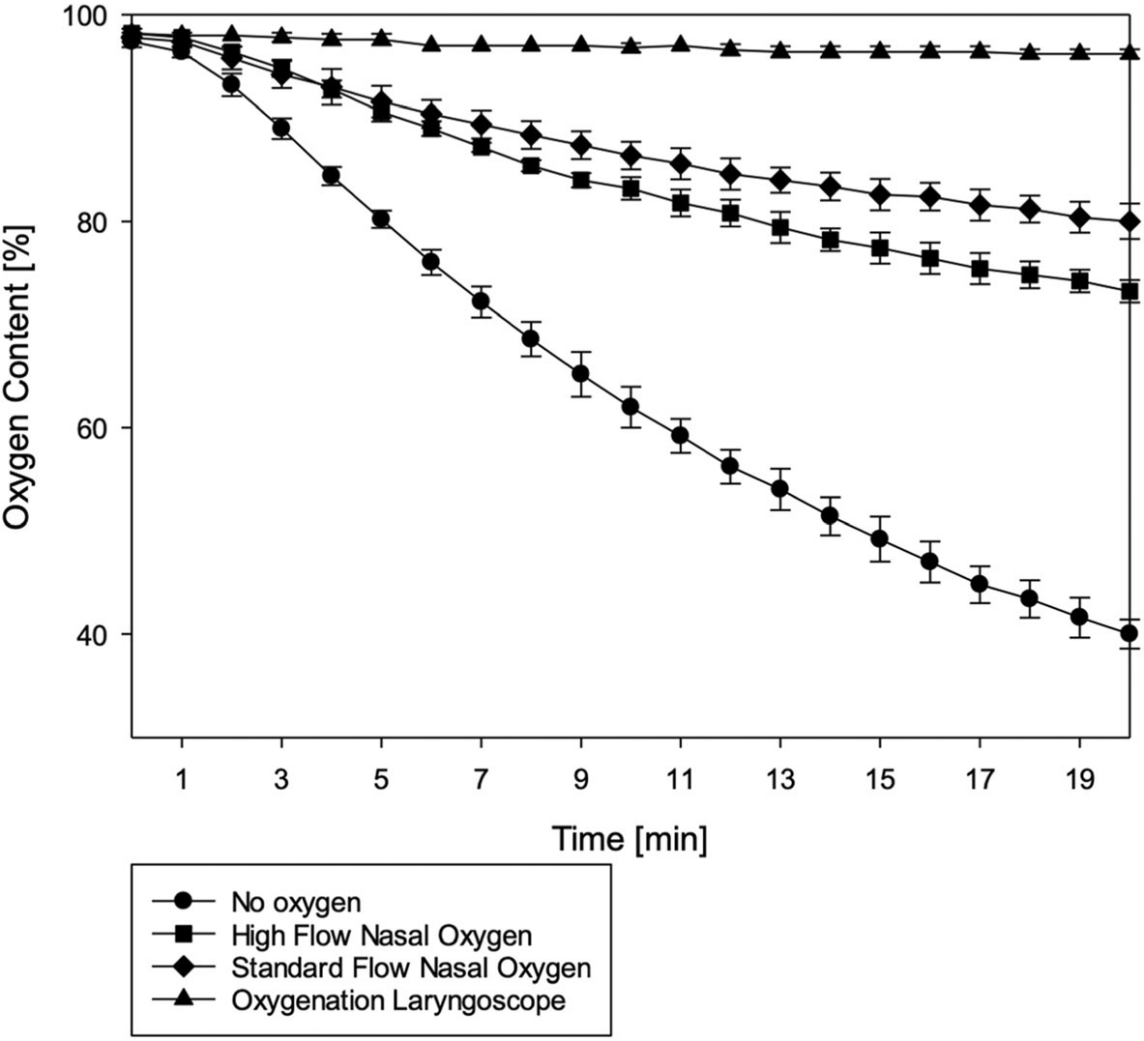
Oxygen concentration in the simulation lung vs. time; *p* < 0.009 between all groups

The detailed course of oxygen content over the time in the different groups is shown in detail in Fig. 3.

## Discussion

Laryngeal oxygen insufflation via oxygenation laryngoscope may provide a simple and rapid further oxygenation strategy in an *unexpected cannot-intubate-cannot-ventilate* scenario. This could provide some extra time for alternative airway management strategies, or to bridge time until a more skilled anaesthetist or a device like a video laryngoscope arrive on scene. It could also provide extra time for suctioning in a soiled airway. The oxygenating laryngoscope is easy to use, and oxygen insufflation can be established within seconds.

In this study, oxygen insufflation via the oxygenating laryngoscope was more effective than conventional nasal oxygen insufflation with 10 l·min^− 1^, as described previously [5]. Not surprisingly, the decrease in oxygen concentration in our simulation lung was delayed by standard nasal oxygen insufflation compared to the control group using air too.

In this model, oxygen insufflation via the oxygenation laryngoscope was more effective than nasal oxygen insufflation at both standard and high gas flows. We have previously attributed a comparable phenomenon to the kinked human airway. Oxygen given via nasal cannula pours into the upper pharynx, where it most likely mixes with nitrogen entering through the manikin’s mouth as in a mixing chamber [6]. In contrast, during deep laryngeal insufflation, oxygen pours out retrogradely through the manikin’s mouth, which is obviously more effective in continuous denitrogenisation of the airway [8]. Also, oxygen insufflation via the oxygenation laryngoscope is simple and does not interfere with intubation efforts.

We were surprised to find that using a high flow system at 20 l·min^− 1^ with 90% oxygen concentration (limited due to technical limitations) resulted in a lower oxygen concentration in the simulation lung than when using the oxygenation laryngoscope or even nasal oxygen insufflation at standard flows with 10 l·min^− 1^ pure oxygen. This is a phenomenon that we cannot completely explain. At higher flow speed oxygen was pouring faster in the upper airway than when using conventional oxygen insufflation at 10 l·min^− 1^, which may have resulted in a more turbulent airflow. Airflows can be described by Reynolds Number (RE). Increasing flow speed can result in a higher RE and thus a more turbulent gas flow. This more turbulent gas flow may increase the inflow of air in the mouth and thus increase mixing with insufflated oxygen in the upper airway. Another possible explanation might be the suctioning of ambient air in the airway at the nostrils where the high flow cannula was placed. At higher gas flows there may be local sub-atmospheric pressures due to the Bernoulli effect, resulting in ambient air being carried along with the insufflated oxygen into the airway. Regardless of the mechanism, higher gas flows must result in higher grades of mixing air in the upper airway with the applied 90% oxygen, since the oxygen content in the simulation lung decreased faster than during standard nasal oxygen insufflation.

This may also be one possible explanation as to why the application of high flow oxygen does not show improved results during the intubation of critically ill patients [9], whereas standard oxygen insufflation for apnoeic oxygenation shows benefits in regard to time until oxygen desaturation [10, 11]. We recommend this should be the subject of specific studies. Further, it may have adverse effects, for example, deep nasal insertion of a cannula poses the risk of nasal bleeding, which could compromise an already difficult airway further [8]. Another disadvantage of high flow nasal oxygenation is the increased technical effort compared to standard oxygen application, since it needs special devices, whereas additional oxygen supply lines can be attached to even standard or video laryngoscopes. This may be even an additional device for preparedness in regard of cannot-ventilate-cannot-intubate scenarios until a surgical airway can be implemented [12]. Further, applying oxygen at lower flows could be even an advantage in regards of aerosol production during the intubation process in times of COVID 19.

We were not able to determine whether further increased gas flows improve results, since due to technical reasons the maximum oxygen concentration in the insufflated gas is 45% at the maximum possible 60 l·min^− 1^. This concentration may be perfectly sufficient in spontaneously breathing patients, but it is not suitable to maintain apnoeic oxygenation. Thus, we did not test this. In contrast to this situation where we suctioned all gases from the simulation lung, in a living organism, nitrogen that has entered the lungs is accumulating there quickly as it does not dissolve in the blood stream and impairs oxygen uptake due to disturbing apnoeic oxygenation. In this regard we aimed to see about 70% oxygen concentration as an acceptable result after 20 min. Due to the accumulation of nitrogen, this would have already resulted in severe hypoxia in a living organism. Thus, this model simulates relative differences between oxygenation methods but does not provide correct values with regard to rate of oxygen concentration decrease in living organisms. Therefore, we point out that a technical simulation will always have limitations. However, as described previously it should be a good method to compare the effects of different methods of oxygen application in a human airway [5].

Human experiments resulting in deliberate desaturation are unethical by any standard, animal experiments are not realistic due to differences of anatomy, and attaching a living organism to a manikin in a hybrid model does not provide any further information. This latter technique would indeed simulate apnoeic oxygenation more precisely but that principle had already been tested over 100 years ago [13]. Thus, our simulation model should be realistic enough and, although a compromise, the best available model to test the formulated hypothesis.

The introduction of video laryngoscopes in clinical routine has changed airway management strategies, as this technology facilitates intubation also in many difficult airway scenarios. Thus, the combination of an oxygenating laryngoscope with video laryngoscopy technology could be a useful invention for future airway management.

## Conclusions

In this technical simulation, oxygenation via laryngoscope was more effective than standard oxygen insufflation via nasal cannula, which was more effective than nasal high flow insufflation of 90% oxygen.

## Data Availability

The datasets analysed during the current study available from the corresponding author on reasonable request. All data generated used or analysed during this study are included in the published article.

## Abbreviations

RE: Reynolds Number
